# Antimicrobial Resistance, Healthcare-Associated Infections, and Environmental Microbial Contamination

**DOI:** 10.3390/healthcare10020242

**Published:** 2022-01-27

**Authors:** Maria Dolores Masia, Marco Dettori

**Affiliations:** 1 Department of Medical, Surgical and Experimental Sciences, University of Sassari, 07100 Sassari, Italy; mdmasia@uniss.it; 2 University Hospital of Sassari, 07100 Sassari, Italy

## 1. Background

In the context of clinical risks, infectious risk, i.e., the probability of contracting an infection during healthcare facilities stays, is the most serious and common adverse effect and/or complication of healthcare. Hospital infections, and more generally, healthcare-associated infections (HAIs), are in fact one of the main problems in terms of morbidity, mortality, and costs that public health is currently facing [1,2].

The ever-increasing knowledge of the risk factors associated with hospitalisation has undoubtedly improved our ability to control HAIs, but the frequency with which they occur has remained more or less constant over the years. This is also due to an increase in the population that is particularly susceptible, which is in turn attributable, at least in part, to positive factors. Indeed, the progressive ageing of the population and the increased survival of patients with highly debilitating pathologies (made possible by the refinement of diagnostic and therapeutic techniques) means that many of today’s patients requiring care are individuals with reduced immune defences and are therefore more exposed to the risk of infection [3,4].

Like all infections, HAIs are the result of the interaction of three elements: the host, the infecting agent, and the environment. In particular, the habitus of the patient has an important influence on the infectious risk. Consequently, those patients most at risk are mainly the elderly, premature, and immunocompromised. The habitus also partly accounts for the type of microorganisms primarily involved. Those responsible for HAIs are not usually conventional pathogens, but mainly saprophytic microorganisms that find ideal conditions for propagation and development in the characteristics of the patients and the care environment. Until the early 1980s, HAIs were mainly caused by Gram-negative bacteria (e.g., *Escherichia coli and Klebsiella pneumoniae*), but subsequently, as a result of antibiotic pressure and the increased use of plastic medical devices, an increase in infections with Gram-positive bacteria and fungi has been observed. Moreover, HAIs are now increasingly caused by microorganisms that are resistant to first-line drugs and are often also multi-resistant, such as MRSA (*Methicillin-Resistant Staphylococcus Aureus*), VRE (*Vancomycin-Resistant Enterococci*), and CPE (*Carbapenemase-Producing Enterobacteria*) [5,6]. In particular, it is estimated that one in three infections in Europe are sustained by antibiotic-resistant microorganisms. Furthermore, there is growing evidence that the environment can act as a reservoir of microorganisms and contribute to their spread [7,8].

Like all infections, HAIs have different modes of transmission: direct, interhuman, and indirect. Of critical importance are the cleanliness of workers’ hands, the use of medical devices and diagnostic and/or therapeutic manoeuvres, contaminated instruments, objects, and solutions, as well as other vehicles and vectors.

Among the various vehicles, air plays a particularly important role. Air is the vehicle through which microorganisms move through the environment, reach surfaces, and settle there, resulting in the risk of exposure through inhalation, contact with contaminated surfaces/objects or ingestion. It has been estimated that about 10% of HAIs are attributable to microbial contamination of the air, i.e., the presence of pathogenic and/or higher-than-acceptable quantities of microorganisms. Furthermore, inappropriate microclimatic conditions can affect the microbial *facies* of the air, leading to the inhalation of microbial aerosols and the deposition of contaminated particles.

The environment’s structures and plants can also play an important role. In this context, water distribution systems and aerosols released from cooling systems, for example, should be considered, as here, microorganisms of purely environmental origin may be present, which find an ideal habitat in man-made water systems (e.g., *Legionella* spp., non-tubercular *Mycobacteria*, amoebas) [8,9,10].

It is clear that control, prevention, and surveillance measures are the best strategies to contain the spread of HAIs [11]. However, despite their high social and economic impact, the surveillance and control systems for these infections and the actions implemented to reduce their effects are inefficient and not homogeneous, and many international studies agree that this failure is due to an insufficient systemic adaptation by healthcare organisations.

National authorities play important leadership roles in developing policies, recommendations, and guidelines in order to provide the necessary qualified personnel, facilitate the implementation of prevention and control practices in health facilities, monitor progress, and give feedback. In this regard, the identification and implementation of elements that characterise an optimal plan for HAI control and prevention are essential. Such elements include: (i) standard precautions (hand hygiene and the use of barrier measures), (ii) the monitoring of environmental microbial contamination and control of environmental sanitisation procedures, (iii) the disinfection and sterilisation of reusable equipment and devices, (iv) the availability of resources (be they human resources, with reference to the patient/staff ratio, or technological resources with regard to the availability of devices or devices effective in reducing the transmission of infections); (v) the constant monitoring of plant structures (e.g., aero-hydraulic and plumbing systems); (vi) the promotion of educational initiatives, as well as specific training, aimed at cultural and behavioural change on the part of health and social service providers and citizens, making best use of the scientific evidence [10,12,13,14,15].

More generally, given that one of the main responsibilities of the mission of healthcare institutions is to provide quality care while guaranteeing the safety of patients and healthcare workers, the surveillance and control of all risk factors present in healthcare facilities must be an essential aspect of care [10,16,17,18].

The in-depth examination of these issues in a dedicated Special Issue, through a multidisciplinary overview, may represent a further tool available to the scientific community to identify the best practices of proven effectiveness in the field for the implementation of decisive strategies aimed at tackling this crucial phenomenon for public health.

## 2. Conclusions

With this in mind, the Special Issue “Antimicrobial Resistance, Healthcare-Associated Infections, and Environmental Microbial Contamination”, published in the journal *Healthcare*, primarily aims to increase the international literature evidence and observations in the field regarding the implementation of standard precautions, the adoption of specific infectious disease precautions, the improvement of staff education and responsibility, environmental monitoring, facility audits, and the appropriate use of antimicrobials.

## Data Availability

Not applicable.
